# Implications of climatic change on sickle cell anemia: A review

**DOI:** 10.1097/MD.0000000000037127

**Published:** 2024-02-09

**Authors:** Emmanuel Ifeanyi Obeagu, Getrude Uzoma Obeagu

**Affiliations:** a Department of Medical Laboratory Science, Kampala International University, Kampala, Uganda; b School of Nursing Science, Kampala International University, Kampala, Uganda.

**Keywords:** adaptation strategies, climate change, disease management, healthcare infrastructure, sickle cell anemia, weather patterns

## Abstract

Sickle cell anemia (SCA) is a hereditary blood disorder characterized by abnormal hemoglobin, causing red blood cells to assume a sickle shape, leading to various complications. Climate change has emerged as a significant global challenge, influencing environmental conditions worldwide. This paper explores the implications of climatic variations on the prevalence, management, and outcomes of SCA. Climate change affects weather patterns, leading to altered temperatures, increased frequency of extreme weather events, and variations in humidity levels. These changes can have a profound impact on individuals living with SCA. High temperatures exacerbate the symptoms of SCA, potentially triggering painful vaso-occlusive crises due to dehydration and increased blood viscosity. Conversely, cold temperatures may induce vaso-occlusion by causing blood vessels to constrict. Changes in rainfall patterns might also affect water accessibility, which is crucial for maintaining adequate hydration, particularly in regions prone to droughts. The management of SCA is multifaceted, involving regular medical care, hydration, and avoiding triggers that could precipitate a crisis. Adverse weather events and natural disasters can disrupt healthcare infrastructure and access to essential medications and resources for SCA patients, especially in vulnerable communities. To mitigate the implications of climatic change on SCA, interdisciplinary strategies are essential. These strategies may include enhancing healthcare systems’ resilience to climate-related disruptions, implementing adaptive measures to address changing environmental conditions, and promoting public awareness and education on managing SCA amidst climate variability. In conclusion, climatic variations pose significant challenges for individuals with SCA, affecting the prevalence, management, and outcomes of the disease.

## 1. Introduction

Sickle cell anemia (SCA), also known as sickle cell disease, is a hereditary condition that alters the shape and function of red blood cells.^[1–3]^ While the disease is primarily caused by a mutation in the HBB gene, environmental factors, including temperature, humidity, and air quality, can exacerbate its effects.^[4]^ With global climatic changes ongoing, the implications for individuals with SCA are increasingly significant.^[5]^ The frequency and intensity of heatwaves are increasing worldwide due to climate change. Elevated temperatures can trigger vaso-occlusive crises, a hallmark of SCA, causing severe pain and organ damage in affected individuals. Additionally, extreme cold weather can lead to vasoconstriction, potentially increasing the risk of sickle cell crisis and tissue damage.^[6–8]^ Climate change can influence regional patterns of precipitation and humidity, affecting the risk of dehydration for individuals with SCA. Dehydration can trigger sickling of red blood cells and lead to painful crises. Changes in water availability may also affect the ability of SCA patients to maintain adequate hydration, potentially influencing their health outcomes.^[9–11]^

Climatic changes can influence air quality and the prevalence of air pollutants, which can impact respiratory health. People with SCA are already at risk for pulmonary complications, and poor air quality may exacerbate these issues.^[12,13]^ Climatic changes can alter the geographic distribution and prevalence of infectious diseases. SCA individuals may be more vulnerable to certain infections, so shifts in disease patterns could have significant implications for their health.^[14,15]^ Health systems need to adapt to the changing health needs of individuals with SCA in a shifting climate. This includes better management of acute crises and long-term care to prevent complications.^[16]^ Public health initiatives aimed at both climate adaptation and SCA management can help individuals with SCA better navigate the challenges posed by climatic change. These initiatives include heat advisories, hydration promotion, and support for staying cool in hot weather.^[17]^

## 2. Climate change and sickle cell anemia

Climate change is a global phenomenon with far-reaching implications for various aspects of human health, including the management and outcomes of genetic diseases such as SCA.^[18]^ SCA is an inherited blood disorder that primarily affects people of African, Mediterranean, and Middle Eastern descent. The disease is characterized by the production of abnormal hemoglobin, leading to the deformation of red blood cells and various health complications. This article explores the intersection of climate change and sickle cell anemia, highlighting the potential impacts of changing environmental conditions on individuals living with this genetic disorder.^[19]^ One of the most direct effects of climate change is the alteration of temperature patterns. Rising global temperatures have led to more frequent and intense heatwaves in many regions. This is of particular concern for individuals with SCA, as extreme heat can trigger vaso-occlusive crises. These crises are painful episodes that occur when the misshapen red blood cells block blood flow, leading to severe pain and organ damage. High temperatures can exacerbate this condition, increasing the frequency and severity of crises.^[20,21]^ Conversely, extreme cold weather can also have adverse effects on individuals with SCA. Cold temperatures can cause vasoconstriction, reducing blood flow and oxygen delivery to tissues. This can result in pain and potentially contribute to sickle cell crisis.^[22]^

Climate change can significantly affect regional patterns of precipitation and humidity. Dehydration is a known trigger for vaso-occlusive crises in individuals with SCA. Changes in water availability and increasing temperatures can lead to an increased risk of dehydration, further exacerbating the condition.^[23,24]^ On the other hand, individuals with SCA are encouraged to stay well-hydrated to prevent crises. Changes in precipitation patterns can make it more challenging for SCA patients to maintain adequate hydration, potentially impacting their health outcomes.^[25]^ Climate change can influence air quality through various mechanisms. Increased temperatures can lead to the formation of ground-level ozone, a harmful air pollutant. Poor air quality can have significant health impacts, especially on individuals with preexisting respiratory conditions. For individuals with SCA, who are already at risk for pulmonary complications, poor air quality can exacerbate their respiratory issues and increase the likelihood of acute health crises.^[26]^ Climate change can alter the distribution and prevalence of infectious diseases. People with SCA are more vulnerable to certain infections due to their weakened immune system. Shifts in disease patterns, such as the expansion of vector-borne diseases into new regions, can pose additional risks to SCA patients.^[27]^ The intersection of climate change and sickle cell anemia presents a complex and multifaceted challenge. While climate change affects everyone, individuals with SCA face unique risks due to their genetic condition. Understanding and addressing these risks is crucial for improving the quality of life and health outcomes for those living with SCA.^[28]^ Efforts to mitigate these risks include heat advisories, promotion of proper hydration, and support for individuals with SCA to manage their condition effectively in changing environmental conditions. Additionally, healthcare systems and providers must adapt to the evolving needs of SCA patients as climate change continues to impact health outcomes worldwide.^[29]^

## 3. Implications of climatic change on sickle cell anemia for healthcare systems

The effects of climate change on health are becoming increasingly apparent, and healthcare systems need to adapt to these changes.^[30]^ This paper discusses the implications of climatic change on SCA for healthcare systems and the measures needed to provide effective care and support for affected individuals. As climate change contributes to more frequent and severe weather events, including extreme heatwaves and cold snaps, healthcare systems are likely to experience an increase in the demand for services related to SCA. These extreme weather events can trigger vaso-occlusive crises, which are painful episodes that require immediate medical attention. Healthcare facilities need to be prepared for spikes inpatient admissions during these extreme weather conditions.^[31]^ Climate change can affect the geographic distribution and prevalence of infectious diseases. Individuals with SCA are already at an increased risk of infections due to their compromised immune systems. Changes in disease patterns, such as the emergence of new vector-borne diseases in previously unaffected areas, can pose additional health risks. Healthcare systems must monitor and respond to these changing disease patterns, ensuring that SCA patients are protected from potentially life-threatening infections.^[32]^ Healthcare infrastructure, including hospitals and clinics, needs to be resilient in the face of climatic change. Increased temperatures and more frequent heatwaves can strain air conditioning and cooling systems, which are crucial for the comfort of SCA patients. Adequate infrastructure resilience is essential to ensure that healthcare facilities remain functional during extreme weather events and that the care provided is not compromised.^[33]^ Healthcare systems should collaborate with public health agencies to develop and implement interventions that address the specific needs of SCA patients in a changing climate. These interventions may include:^[34]^ Heat advisories and early warning systems to alert individuals with SCA and their caregivers about impending heatwaves. Education and outreach programs to promote proper hydration and temperature regulation among SCA patients. Telehealth services to provide remote care and support during extreme weather events when transportation to healthcare facilities may be challenging. Healthcare systems should emphasize long-term care and disease management for SCA patients. Climate change will continue to impact the health of individuals with SCA over their lifetimes. Healthcare providers should work with SCA patients to develop individualized care plans that consider their unique vulnerabilities and risks related to changing environmental conditions.^[35]^ The implications of climatic change on sickle cell anemia are significant and multifaceted. Healthcare systems need to prepare for increased healthcare demand during extreme weather events and adapt their infrastructure to remain functional and resilient. Collaborative efforts with public health agencies and tailored interventions are essential to support individuals with SCA in a changing climate. Ultimately, healthcare systems must prioritize long-term care and disease management to ensure that SCA patients can lead healthy lives despite the challenges posed by climatic change.^[36]^

## 4. Pathophysiological implications of climate change

SCA is a genetic disorder characterized by abnormal hemoglobin that causes red blood cells to become rigid and sickle-shaped, leading to various complications. Climate change can have several pathophysiological implications specifically for individuals living with sickle cell anemia. Individuals with sickle cell disease are more vulnerable to infections, especially from certain bacteria and viruses. Changes in climate patterns, such as increased temperatures or altered rainfall patterns, can affect the prevalence and distribution of infectious diseases. This could potentially increase the risk of infections like malaria, which can be particularly severe in individuals with sickle cell anemia.^[37–42]^

Changes in temperature and humidity can influence the frequency and severity of vaso-occlusive crises, which are episodes of severe pain caused by the blockage of blood vessels by sickled red blood cells.^[43]^ Extreme temperatures, whether hot or cold, can trigger these painful crises, making climate change-related temperature fluctuations a concern for individuals with sickle cell anemia. Higher temperatures associated with climate change can lead to increased rates of dehydration. Dehydration is a risk factor for sickle cell crises, as it contributes to the sickling of red blood cells and vaso-occlusion. Heat stress can exacerbate this risk, potentially leading to more frequent and severe pain crises.

Climate change-induced extreme weather events, such as hurricanes, floods, or prolonged heatwaves, can disrupt healthcare infrastructure and access to essential medications and treatments for individuals with sickle cell anemia.^[43]^ Disruptions in healthcare services can lead to inadequate management of the disease and an increased risk of complications. Changes in climate can impact agricultural patterns, affecting food availability and nutritional quality. Individuals with sickle cell anemia require a well-balanced diet to manage their condition. Disruptions in food supply due to climate change-related factors can potentially affect their nutritional intake and overall health. Individuals with chronic illnesses like sickle cell anemia may experience increased stress and anxiety during extreme weather events or in the face of climate change-related challenges. Mental health impacts can indirectly influence the management of the disease and overall well-being. It is crucial to recognize the potential implications of climate change on individuals with sickle cell anemia and take proactive measures to address these challenges. This includes ensuring access to healthcare, medications, and supportive services, as well as raising awareness among both patients and healthcare providers about the intersection of climate change and the management of sickle cell disease.

## 5. Prevention measures

Preventing the adverse effects of climate change on individuals with sickle cell anemia involves a combination of general climate change mitigation strategies and targeted measures to manage the specific health risks faced by these individuals. Ensure consistent access to healthcare services, including regular checkups, medications, and treatments for individuals with sickle cell anemia.^[44]^ Establish emergency preparedness plans within healthcare systems to address potential disruptions caused by climate change-related events. Educate individuals with sickle cell anemia and their caregivers about the potential impacts of climate change on their health and the specific measures they can take to mitigate risks.^[45]^ Provide information about managing dehydration, heat stress, and the prevention of pain crises during extreme weather conditions. Encourage individuals with sickle cell anemia to stay hydrated and avoid prolonged exposure to extreme temperatures. Advising them to stay in cool environments and use air conditioning, when possible, can help prevent heat-related complications. Educate patients and caregivers on recognizing early signs of heat-related illnesses and the appropriate actions to take in such situations.

Emphasize the importance of preventive measures against vector-borne diseases, especially malaria, which can significantly impact individuals with sickle cell anemia. This includes the use of insect repellents, bed nets, and appropriate medications or vaccines where available. Implement environmental measures to reduce mosquito breeding sites and control vector populations in affected areas. Promote a healthy and well-balanced diet to support the nutritional needs of individuals with sickle cell anemia. Access to fresh and nutritious food is vital. Emphasize the importance of adequate hydration to prevent dehydration, which can trigger sickle cell crises. Encourage regular intake of fluids, especially during hot weather.^[45]^

Provide psychosocial support and resources to help individuals with sickle cell anemia cope with the stress and anxiety associated with climate change-related challenges and potential health impacts. Foster community support groups or networks that can provide emotional support and share coping strategies. Support research initiatives focused on understanding the intersection of climate change and sickle cell anemia to develop targeted interventions and strategies. Advocate for policies that prioritize the healthcare needs of individuals with chronic conditions like sickle cell anemia within climate change adaptation and mitigation efforts.

## 6. Public health interventions for the impact of climatic change on sickle cell anemia

The intersection of climatic change and SCA presents unique challenges, as changing environmental conditions can exacerbate the symptoms and health risks associated with the disease.^[46]^ To address these challenges, public health interventions are crucial in supporting individuals with SCA in adapting to a changing climate. Implement heat advisory systems to provide early warnings to individuals with SCA and their caregivers about impending heatwaves. These advisories should include information on how to stay cool, hydrated, and avoid overheating, which can trigger vaso-occlusive crises. Launch educational campaigns targeting both individuals with SCA and the general public. These campaigns should emphasize the importance of maintaining proper hydration, regulating body temperature, and recognizing the signs of a crisis. Provide guidance on what actions to take during extreme weather conditions. Expand telehealth services to ensure that individuals with SCA have access to medical advice and support during extreme weather events. Telehealth can be vital when transportation to healthcare facilities is challenging, allowing patients to consult with healthcare providers remotely. Create cooling centers in areas with a high prevalence of SCA patients. These centers should offer a safe, air-conditioned environment for individuals to escape extreme heat and cool down. Collaboration with local governments and community organizations can help establish and maintain these facilities. Provide access to clean drinking water in public spaces, especially during heatwaves. Individuals with SCA need to stay well-hydrated to prevent crises, and easy access to water sources can encourage proper hydration. Engage in community outreach programs to identify and support individuals with SCA in vulnerable communities. These programs can help connect patients with essential resources, such as cooling devices, financial assistance, and healthcare information. Collaborate with emergency response agencies to ensure that individuals with SCA are identified and prioritized during extreme weather events. Create plans to address their specific needs, including access to medical care, transportation, and emergency medications. Promote genetic counseling and testing to identify individuals at risk of carrying the sickle cell gene. Awareness and early diagnosis can help individuals make informed decisions about family planning and reduce the prevalence of the disease in vulnerable populations. Advocate for public policies that address the unique challenges faced by individuals with SCA in the context of climate change. This includes advocating for the development and implementation of heat action plans, air quality regulations, and support for research into the intersection of climatic change and SCA. Support research initiatives that explore the complex relationship between climatic change and SCA. Data collection and analysis can help healthcare and public health professionals develop evidence-based interventions and policies to protect individuals with SCA in a changing climate.^[47–49]^ Public health interventions are essential to protect individuals with sickle cell anemia from the adverse effects of climatic change. These interventions should focus on education, access to healthcare, and support during extreme weather events. By addressing the specific needs of individuals with SCA in a changing climate, public health efforts can contribute to improved quality of life and better health outcomes for this vulnerable population.

## 7. Conclusion

The implications of climatic change on individuals with sickle cell anemia are multifaceted and require careful consideration. As the world continues to grapple with the consequences of a changing climate, it is vital to recognize the unique challenges and risks that SCA patients face. Comprehensive efforts are needed to protect their health, ranging from individual self-care practices to public health interventions and healthcare system adaptations. Addressing the intersection of climate change and sickle cell anemia is essential for improving the quality of life and outcomes for those affected by this genetic disorder.

## Author contributions

**Conceptualization:** Emmanuel Ifeanyi Obeagu.

**Methodology:** Emmanuel Ifeanyi Obeagu.

**Supervision:** Emmanuel Ifeanyi Obeagu.

**Validation:** Emmanuel Ifeanyi Obeagu.

**Visualization:** Emmanuel Ifeanyi Obeagu.

**Writing – original draft:** Emmanuel Ifeanyi Obeagu, Getrude Uzoma Obeagu.

**Writing – review & editing:** Emmanuel Ifeanyi Obeagu, Getrude Uzoma Obeagu.
